# Boosting with Subtype C CN54rgp140 Protein Adjuvanted with Glucopyranosyl Lipid Adjuvant after Priming with HIV-DNA and HIV-MVA Is Safe and Enhances Immune Responses: A Phase I Trial

**DOI:** 10.1371/journal.pone.0155702

**Published:** 2016-05-18

**Authors:** Agricola Joachim, Asli Bauer, Sarah Joseph, Christof Geldmacher, Patricia J. Munseri, Said Aboud, Marco Missanga, Philipp Mann, Britta Wahren, Guido Ferrari, Victoria R. Polonis, Merlin L. Robb, Jonathan Weber, Roger Tatoud, Leonard Maboko, Michael Hoelscher, Eligius F. Lyamuya, Gunnel Biberfeld, Eric Sandström, Arne Kroidl, Muhammad Bakari, Charlotta Nilsson, Sheena McCormack

**Affiliations:** 1 Department of Microbiology and Immunology, Muhimbili University of Health and Allied Sciences, Dar es Salaam, Tanzania; 2 Department of Microbiology, Tumor and Cell Biology, Karolinska Institutet, Stockholm, Sweden; 3 National Institute for Medical Research-Mbeya, Medical Research Center, Mbeya, Tanzania; 4 Department of Infectious Diseases and Tropical Medicine, Medical Center of the University of Munich (LMU), Munich, Germany; 5 Medical Research Council Clinical Trials Unit, University College London, London, United Kingdom; 6 German Center for Infection Research (DZIF), partner site Munich, Munich, Germany; 7 Department of Internal Medicine, Muhimbili University of Health and Allied Sciences, Dar es Salaam, Tanzania; 8 Department of Surgery and Molecular Genetics and Microbiology, Duke University Medical Center, Durham, North Carolina, United States of America; 9 The Military HIV Research Program, Walter Reed Army Institute of Research, Silver Spring, Maryland, United States of America; 10 The Military HIV Research Program, The Henry M. Jackson Foundation for the Advancement of Military Medicine, Bethesda, Maryland, United States of America; 11 Imperial College London, London, United Kingdom; 12 Venhälsan, Karolinska Insitutet at Södersjukhuset, Stockholm, Sweden; 13 The Public Health Agency of Sweden, Solna, Sweden; 14 Department of Laboratory Medicine, Karolinska Institutet Huddinge, Stockholm, Sweden; The George Washington University School of Medicine and Health Sciences, UNITED STATES

## Abstract

**Background:**

A vaccine against HIV is widely considered the most effective and sustainable way of reducing new infections. We evaluated the safety and impact of boosting with subtype C CN54rgp140 envelope protein adjuvanted in glucopyranosyl lipid adjuvant (GLA-AF) in Tanzanian volunteers previously given three immunizations with HIV-DNA followed by two immunizations with recombinant modified vaccinia virus Ankara (HIV-MVA).

**Methods:**

Forty volunteers (35 vaccinees and five placebo recipients) were given two CN54rgp140/GLA-AF immunizations 30–71 weeks after the last HIV-MVA vaccination. These immunizations were delivered intramuscularly four weeks apart.

**Results:**

The vaccine was safe and well tolerated except for one episode of asymptomatic hypoglycaemia that was classified as severe adverse event. Two weeks after the second HIV-MVA vaccination 34 (97%) of the 35 previously vaccinated developed Env-specific binding antibodies, and 79% and 84% displayed IFN-γ ELISpot responses to Gag and Env, respectively. Binding antibodies to subtype C Env (included in HIV-DNA and protein boost), subtype B Env (included only in HIV-DNA) and CRF01_AE Env (included only in HIV-MVA) were significantly boosted by the CN54rgp140/GLA-AF immunizations. Functional antibodies detected using an infectious molecular clone virus/peripheral blood mononuclear cell neutralization assay, a pseudovirus/TZM-bl neutralization assay or by assays for antibody-dependent cellular cytotoxicity (ADCC) were not significantly boosted. In contrast, T-cell proliferative responses to subtype B MN antigen and IFN-γ ELISpot responses to Env peptides were significantly enhanced. Four volunteers not primed with HIV-DNA and HIV-MVA before the CN54rgp140/GLA-AF immunizations mounted an antibody response, while cell-mediated responses were rare. After the two Env subtype C protein immunizations, a trend towards higher median subtype C Env binding antibody titers was found in vaccinees who had received HIV-DNA and HIV-MVA prior to the two Env protein immunizations as compared to unprimed vaccinees (p = 0.07).

**Conclusion:**

We report excellent tolerability, enhanced binding antibody responses and Env-specific cell-mediated immune responses but no ADCC antibody increase after two immunizations with a subtype C rgp140 protein adjuvanted in GLA-AF in healthy volunteers previously immunized with HIV-DNA and HIV-MVA.

**Trial Registration:**

International Clinical Trials Registry PACTR2010050002122368

## Introduction

A vaccine against HIV is widely considered the most effective and sustainable way of reducing new infections [1]. Of the six HIV vaccine efficacy trials conducted to date, only one has demonstrated efficacy [2–7]. In 2009, the RV144 “Thai” trial reported 31.2% protection against HIV infection, with no associated impact on HIV viral load or CD4^+^ T cell count in vaccinated infected individuals. This placebo controlled trial conducted in a low incidence, largely heterosexual population in Thailand randomized 16402 men and women to receive four immunizations with canarypox (ALVAC–HIV vCP1521) given twice on its own and then twice more in combination with a monomeric envelope protein (AIDSVAX B/E gp120) in alum [7]. The modest reduction of acquisition of HIV infection is thought to correlate with non-neutralizing antibodies, most likely associated with antibody-dependent cellular cytotoxicity (ADCC) [8–10].

The HIVIS/TaMoVac consortium has optimized the delivery of multisubtype HIV-DNA and HIV-MVA vaccine candidates in a series of trials, and have shown them to be safe and potent T-cell and B-cell immunogens [11–15]. The recent TaMoVac 01 trial focussed on the optimization of needle free priming and included 120 healthy men and women in Tanzania. The volunteers were randomized to receive (i) 600 μg or 1000 μg of HIV-DNA as either (ii) pooled or separate Env and Gag encoding plasmid pools intradermally (ID) via the needle free Zetajet device [16]. There were no vaccine related safety concerns and no significant differences between the groups with respect to the magnitude of IFN-γ ELISpot responses or the proportion of individuals who responded (87–97%). Similarly, there was no significant difference between the groups in terms of binding antibody responses (83–97% responders although the titers were low. The simplified regimen offered clear practical and logistical advantages.

An Env protein gp140 (gp120 plus the external domain of gp41) from a subtype C envelope clone p97CN54 (CN54rgp140) has been given to HIV negative women in Europe, formulated in a vaginal gel in MucoVac1 [17], and via the parenteral, nasal and vaginal routes in MucoVac2 [18]. In MucoVac2, nine women received three immunizations of 100 μg of the CN54rgp140 protein given IM with glucopyranosyl lipid A (GLA-AF) over eight weeks, with a subset of five individuals receiving two further immunizations 12–24 weeks later. The adjuvant, GLA-AF is a synthetic monophosphoryl lipid A (MPL)-like molecule which has shown to be a potent activator of dendritic cells *in vitro* [19]. The vaccines were immunogenic when administered parenterally, eliciting systemic specific IgG antibodies in 9/9 (100%) and cervico-vaginal antibodies to CN54rgp140 in 4/9 (44%) vaccine recipients [18].

The results of the RV144 trial reinvigorated interest in the role of non-neutralizing antibodies and the benefits of combining immunogens such as pox viruses and envelope proteins. The HIVIS/TaMoVac consortium had access to the CN54rgp140 protein formulated in GLA-AF through partnership with the UK HIV Vaccine Consortium and we were keen to assess the impact of additional boosting of TaMoVac 01 vaccinees with this Env protein. We hypothezised that this combination would be more potent than the alum adjuvanted AIDSVAX used in RV144. Vaccinees who had received their first immunization 58 weeks or more and had completed the schedule in the TaMoVac 01 trial were invited to receive two further immunizations with 100 μg CN54rgp140 adjuvanted with 5 μg GLA-AF, four weeks apart to assess the safety and immunogenicity of this combination regimen.

## Materials and Methods

### Ethics statement

Ethical approval was obtained from the institutional review boards of the Muhimbili University of Health and Allied Sciences (MUHAS), and the Mbeya Medical Research Ethics Committee. The Tanzanian National Institute for Medical Research (NIMR), serving as the National Health Research Ethics Committee, and the Regional Ethics Committee in Stockholm, Sweden also approved the study. The Tanzania Food and Drugs Authority (TFDA) approved the candidate CN54rgp140/ GLA-AF vaccine for use in humans in Tanzania. This study was conducted according to the principles of International Council of Harmonization and Good Clinical Practice guidelines (ICH-GCP). All participants were provided with an information sheet and were recruited after having signed the study informed consent form.

### Study design and population

This study built upon the TaMoVac 01 trial [17] and was submitted as an amendment to the TaMoVac protocol. The amended study protocol and CONSORT checklist are available as supporting information; see S1 Protocol and S1 CONSORT Checklist. The trial is registered at the World Health Organization International Clinical Trials Registry with registration number PACTR2010050002122368 that is available at http://apps.who.int/trialsearch/Trial2.aspx?TrialID=PACTR2010050002122368.

**Fig 1:**
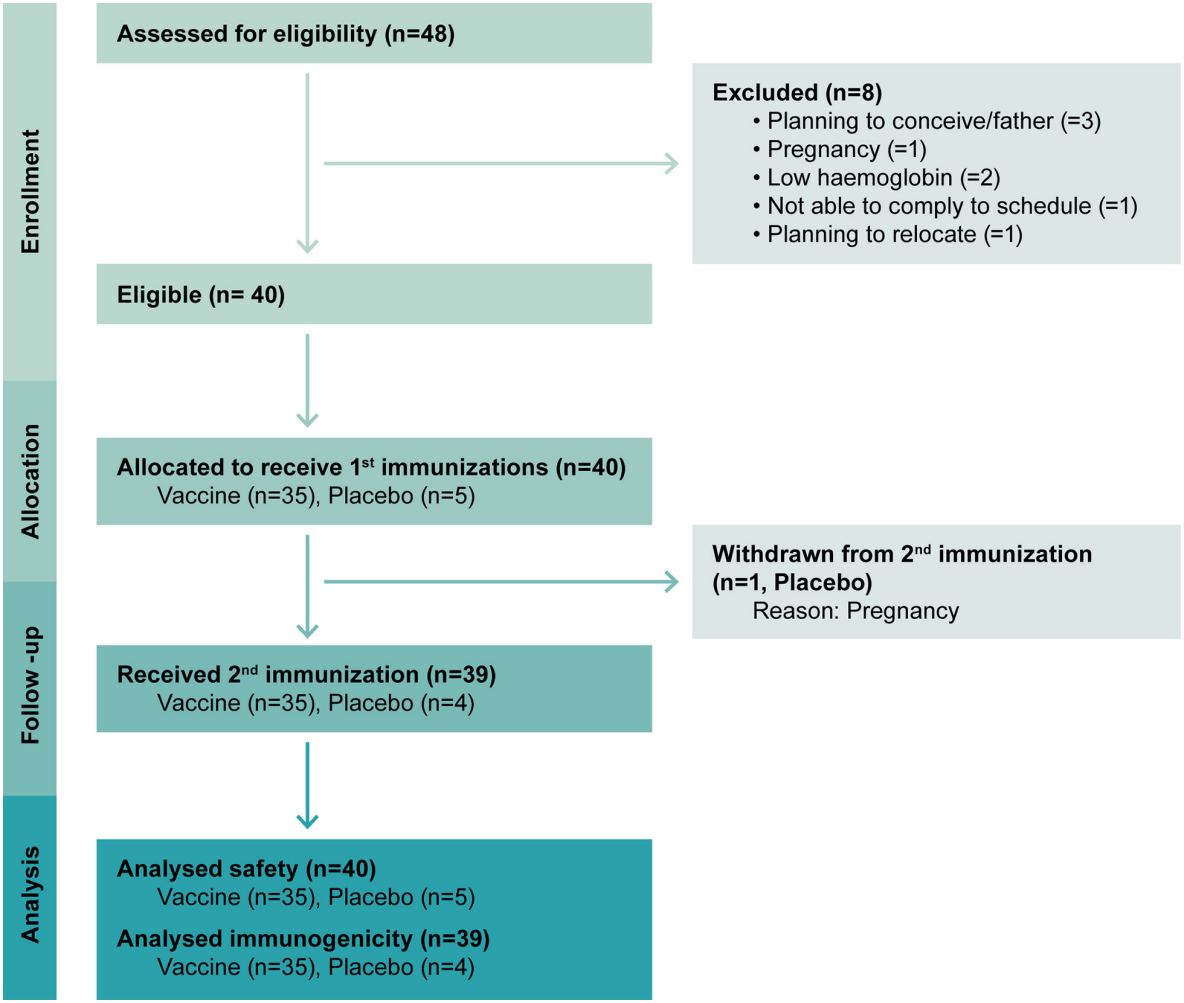
The number of individuals screened, randomized, allocated and withdrawn from the trial.

The TaMoVac 01 trial encluded 120 HIV non-infected, healthy volunteers between March 2010 and June 2011 from two centers in Tanzania: the Muhimbili University of Health and Allied Sciences (MUHAS) in Dar es Salaam, and the National Institute for Medical Research (NIMR)-Mbeya Medical Research Center (NIMR-MMRC) in Mbeya. TaMoVac 01 participants received 7 DNA plasmids encoding HIV-1 subtypes A, B, and C (HIV-DNA) at weeks 0, 4 and 12 intrademally (ID) using the Zetajet device and were boosted intramuscularly (IM) with viral vector vaccine HIV-MVA-CMDR expressing CRF01_AE at weeks 30 and 46. The antigens included in the HIV-DNA and HIV-MVA vaccines are shown in S1 Table. The plasmids were delivered in combined or separated pools (Pool 1: EnvABC/RevB, Pool 2 GagAB/RTmutB) [16]. Immunizations were active or placebo (sterile saline) in a 9:1 ratio. The last TaMoVac 01 study visit was completed in June 2012 (Fig 1 and Table 1).

**Table 1:** Randomized study groups, doses and routes of immunization.

TaMoVac 01 regimen [detailed in 16]				Amendment	
Vaccine	N	HIV-DNA ID	HIV-MVA IM	N	Protein IM
**Time point**		weeks 0, 4 and 12	weeks 30 and 46		Two immunizations with 4 weeks interval after week 58
**Group I**	36	600 μg (combined pools)	10^8^ pfu	10	100 μg CN54gp140 5 μg GLA-AF
**Group II**	36	600 μg (separate pools)	10^8^ pfu	11	100 μg CN54gp140 5 μg GLA-AF
**Group III**	36	1000 μg (separate pools)	10^8^ pfu	14	100 μg CN54gp140 5 μg GLA-AF
**Group IV**	12	Saline ID 2 or 5 x 0.1 ml	Saline IM	5	100 μg CN54gp140 5 μg GLA-AF

N: number of participants. Combined pools refer to a combination of pool 1 (EnvABC/RevB) and pool 2 (GagAB/RTmutB) and separated pools refer to separate administration of pool 1 and 2 into the left and right arm, respectively.

We aimed to enrol 40 TaMoVac 01 trial participants who had received their first HIV-DNA/placebo vaccination at least 58 weeks previously and had received the three HIV-DNA/placebo and two HIV-MVA/placebo immunizations. Individuals were invited to discuss participation and interested volunteers were required to sign an additional informed consent form before screening. Participants were not allowed to take part if they were found to be HIV infected, pregnant, or suffering from any clinically relevant medical condition, had laboratory abnormalities or used immunosuppressive medication. Participants were required to use an effective method of contraception throughout the study period.

### Immunogens and vaccinations

CN54rgp140 is a recombinant subtype C Env protein derived from a Chinese viral isolate 97CM001, clone p97CN54 (S1 Table) [20, 21]. The protein was manufactured using the mammalian CHO cell expression system. It comprises 634 amino acids, and has been shown to be immunogenic in non-human primates and other animal models [22, 23]. The recombinant protein is uncleaved and contains the native sequence (REKR)-reported to be highly cleavage resistant, and contains no additional stabilization mutations such as SOSIP. The identity of the product was confirmed by mass spectrometric analysis of tryptic fragments and stability validated even when kept at room temperature (D. Katinger personal communication, www.polymun.at). CN54rgp140 was manufactured to GMP specifications by Polymun Scientific, Vienna, Austria (accession number AF286226) and purchased by Imperial College London and provided under a Material Transfer Agreement.

GLA-AF is an aqueous formulation containing glucopyranosyl lipid A, a completely synthetic monophosphoryl lipid A (MPL)-like molecule [24]. Both GLA and MPL adjuvants are potent stimulators of the innate immune system, mobilizing antigen presenting cells via binding and activation of toll-like receptor 4. GLA-AF was manufactured to GMP by Infectious Disease Research Institute (IDRI, Seattle, USA), purchased by Imperial College and provided under a Material Transfer Agreement.

CN54rgp140 and GLA-AF were mixed prior to administration IM in a dose of 100 μg gp140/ 5 μg GLA-AF into the deltoid muscle of the left arm at enrolment to the amended protocol and four weeks later.

### Safety assessments

Safety assessments for solicited local and systemic, non-solicited and laboratory adverse events were performed as previously described [16]. Safety assessments were performed at each visit using an open question on adverse events. Information on adverse events recognized to be associated with licensed vaccines was collected on diary cards by participants for one week following each vaccination. These included local (pain, redness, swelling and induration) and general (fever, headache, malaise, chills, nausea, vomiting, myalgia and arthralgia) events. Routine laboratory parameters (full blood count, ALT, direct and indirect bilirubin, random blood glucose and creatinine) were collected at one week after each immunization as well as after four and eight weeks following the final immunization. The clinical and laboratory events were graded for severity as mild, moderate, severe or life threatening based on the DAIDS toxicity scale (Division of AIDS, National Institutes of Health) [25] except for neutropenia which was based on the local reference ranges [26]. HIV infection was regarded as a grade four event according to the study protocol. Each of the clinical events was evaluated for a relationship to vaccine and classified as not related, probably not related, possibly related, probably related and definitely related to the study products.

Urinalysis, pregnancy and HIV tests were performed at screening, on the day of each vaccination and during the final visit. All women were required to have a negative pregnancy test at screening and prior to each vaccination. Participants who were HIV infected or pregnant were stopped from further vaccination but were followed up until the end of the trial or post-delivery for pregnant women. All safety laboratory tests were performed at the Department of Microbiology and Immunology at MUHAS or at MMRC main laboratories. These two laboratories implement strict internal quality control programs and participate in external proficiency testing programs including College of American Pathologists (CAP), United Kingdom National External Quality Assurance Scheme (UKNEQAS) and USA Virology Quality Assurance (VQA).

### Immunological assessments

#### Binding IgG antibody responses

The median titer and estimated concentrations of subtype C CN54gp140-specific IgG antibodies was measured in sera/plasma samples, using enzyme linked immunosorbent assays (ELISAs). ELISA plates (Greiner, Kremsmünster, Austria) were coated with 50 μL per well of anti-Human κ and anti-Human λ capture antibodies mixed at a ratio of 1:1 (SouthernBiotech, Birmingham, USA) or CN54gp140 recombinant protein (homologous to the immunogen) at 1 μg/mL diluted in 1 X DPBS (Thermo Fischer Scientific, Waltham, USA). After incubation overnight at 4°C, plates were washed four times with wash buffer (PBS containing 0.05% Tween 20 (Sigma, St Louis, USA) and then blocked by incubation with 200 μL of PBS containing 1% BSA (Sigma, St Louis, USA) and 0.05% Tween 20 for one hour at 37°C. The standard curve was generated from a serial 5-fold dilution of purified human IgG starting at 1 μg/mL (Sigma). Samples were serially diluted 3-fold starting at 1:100 through to 1:72900. Pooled sera served as controls (NIBSC high and low) and were used neat and at a dilution of 1:100. After blocking and washing the plates four times, 50 μL of the relevant standard, sample or control was added and incubated for an hour at 37°C before washing four times using wash buffer as before. Goat anti-Human IgG-HRP detection antibody (50 μL, Sigma) was added at a dilution of 1:10000 diluted in assay buffer and incubated for 1 hour at 37°C. After final washing, 50 μL of TMB (KPL, Gaithersburg, USA) was added to each well and the plate was incubated for exactly 5 minutes at room temperature in the dark before stopping with 50 μL per well of TMB stop solution (KPL, Gaithersburg, USA). Absorbancies were read immediately at 450 nm using a plate-reader (Tecan, San Jose, USA). A sample was considered as positive in a dilution 1:100 or 1:300 if the absorbance value was more than twice that of the pre-immunization sample at 1:100 or 1:300 dilutions, respectively. A sample was considered positive in a dilution >1:300 if the absorbance value was more than twice the mean of the pre-immunization sample run at a 1:300 dilution. The results were reported as reciprocal end-point titers.

Quantitative results were derived after interpolation of sample values according to standard curve values used for the generation of a linear trendline with r^2^≥0.975.

Binding antibodies to subtype B gp160 were tested as detailed previously using a three-fold dilution series [14]. Micro titer plates (Nunc Maxisorp) were coated with 0.5 μg/mL of subtype B gp160 protein (HIV-1 IIIB, Advanced Biotechnologies Inc, Columbia, MD, USA). End point titers were determined as described above for the subtype C gp140 ELISA.

Binding antibodies to subtype E 93TH975 gp120 recombinant protein (NIH AIDS Research and Reference Reagents program, Division of AIDS, NIAD, Germantown, USA) using 1 μg/mL for coating were determined as described above for the subtype C gp140 ELISA.

#### PBMC neutralization assay

A PBMC based assay, using infectious molecular clones (IMCs) carrying the luciferase gene from *Renilla reneformis* (LucR) as a reporter, was used for measuring neutralization antibody activity [27] as previously described [15]. The IMCs used were SF162 subtype B, GS015 subtype C and CM235 CRF01_AE. The percent neutralization of the post-vaccination serum was calculated based on the level of virus growth in the presence of the same dilution of pre-vaccination serum and neutralization values greater than 50% were considered positive.

#### TZM-bl pseudovirus neutralization assay

Neutralizing antibodies were measured using pseudovirus and a luciferase-based assay in TZM-bl cells as previously described [13]. The preudovirus used included SF162 subtype B, GS015 subtype C and CM235 CRF01_AE.

#### ADCC-luciferase assay

An ADCC assay employing Env.IMC.LucR virus-infected cells as targets [9] was used as previously described [15]. The Env-IMC-LucR viruses used were subtype B IMC_SF162_, (Gen Bank accession no EU123924), CRF01_AE IMC_CM235_ (Gen Bank accession no. AF259954.1) and 1086.c IMC (Gen Bank accession no. FJ444395). ADCC activity was measured as the percent of loss of luciferase activity observed in the presence of serum. The ADCC-mediating antibody titer was defined as the reciprocal of the highest dilution indicating a positive specific killing (>15% specific killing activity) after background subtraction.

#### IFN-γ ELISpot assay

IFN-γ ELISpot was performed on freshly isolated PBMCs using the h-IFN-gamma ELISpot PLUS kit in a two-step detection system (Mabtech, Nacka, Sweden) as previously described [16]. Results were expressed as spot forming cells (SFC) per million PBMC. ELISpot responses were considered positive if the number of spot-forming cells was >4 times the background and baseline value and >55 SFC/10^6^ PBMCs. Data were excluded from analyses if the background responses in medium wells exceeded 60 per million PBMCs.

#### Lymphoproliferation assay (LPA)

Tritiated [^3^H]-thymidine LPA was performed as described previously [12]. T cell proliferation was reported as a stimulation index (SI), determined by dividing the mean counts per minute of the antigen-stimulated wells by the mean of the unstimulated control wells. A SI >6 was considered positive, based on the mean reactivity of 57 healthy Tanzanian volunteers.

### Outcomes

The primary safety endpoint was defined as any grade three and above clinical or laboratory adverse event that occurred after the first immunization up until eight weeks from the last immunization. The primary immunological end point was binding antibodies to subtype C CN54rgp140 measured four weeks after the last vaccination. T-cell responses to vaccine antigens, binding antibody responses to other subtypes and functional antibody responses were also measured four weeks after the final vaccination.

### Statistical methods

Clinical and laboratory safety data were recorded in study specific case report forms and entered twice into the study database which was programmed in SQL. Discrepancies between the data records were resolved before the data files were extracted for analysis. Data were exported directly into Excel from the ELISpot reader. The safety analysis dataset included all solicited, non-solicited and routine laboratory data that were collected after the first vaccination up to eight weeks after the second CN54rgp140/ GLA-AF vaccination. The solicited and non-solicited events were summarized according to the maximum grade of severity reported. Laboratory events were included if they were new or had increased in grade and summarized by grade. The immunological data were analysed using GraphPad Prism version 6 (GraphPad Software, Inc., La Jolla, CA,USA). The Mann-Whitney test was used to compare the magnitude of immune responses before and after the additional immunisations with CN54rgp140/GLA-AF. A two sided p-value of <0.05 was considered to be statistically significant.

## Results

### Demographics, screening and inclusion

Between June and the end of August 2012, 48 volunteers who had participated in the TaMoVac 01 trial were screened and 40 who met the eligibility criteria proceeded to receive two doses of CN54rgp140/GLA-AF. Among these, 21 (52.5%) individuals were from the MMRC and 19 (47.5%) from MUHAS. The mean age was 25 years (range 18 to 39), and 25 (62.5%) were males. Thirty-five (87.5%) participants were previously randomized to one of the vaccine groups in TaMoVac 01 (N = 10 Group I, N = 11 Group II, N = 14 Group III) and the remaining 5 (12.5%) had received placebo (Group IV). Baseline characteristics did not significantly differ between previous TaMoVac 01 group assignments, except that there was a predominance of males in group III (S2 Table). Thirty-nine participants received both vaccinations with a four week interval between August to October 2012. One participant (previously placebo recipient) did not receive the second immunization due to pregnancy. The median interval between the first HIV-DNA and first CN54rgp140/GLA immunization was 103 weeks (range 68 to 114 weeks), and between the last HIV-MVA and first CN54rgp140/GLA immunization it was 60 weeks (range 31 to 70 weeks).

### Safety and tolerability

Solicited local, systemic and laboratory events are presented in S3 Table. In summary 32/40 (80%) vaccinated participants reported a local solicited event and 21 (52.5%) a solicited systemic adverse event that started within one week of an immunization. The majority of these events were mild, however, three participants reported moderate local pain and two participants reported moderate general malaise partially associated with moderate headache, arthralgia and/or myalgia. Twenty-nine laboratory adverse events were recorded in 19/40 (47.5%) participants; 14 (48%) events were detected at safety visits within one week after an immunization. These were mostly mild (N = 24, 83%) or moderate (N = 4, 14%), most frequently presenting as asymptomatic neutropenia and clinically insignificant hypoglycaemia. Three participants experienced mild and transient ALT elevation after the second immunization. Severe, but asymptomatic, hypoglycaemia (glucose <2.24 mmol/L) was detected in one participant with pre-existing moderate hypoglycaemia seven days after the first CN54rgp140/GLA immunization. Only eight non-solicited adverse events were reported during the 12 weeks observation period in 6/40 (15%) participants. All of these were mild or moderate and included infectious diseases in four cases (malaria, tonsillitis or flu like illness), headache in two cases and musculo-skeletal problems in another two cases. None was considered related to the vaccinations. No HIV infection occured.

### Immunological outcomes

#### Binding IgG antibody responses

**Fig 2:**
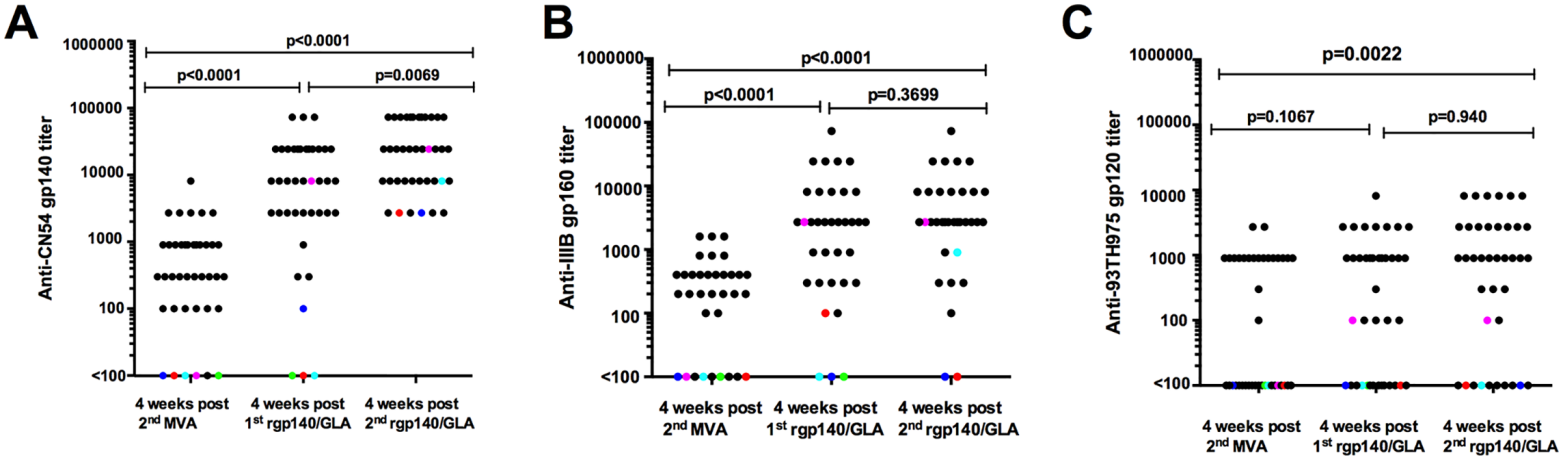
Binding antibody responses to three subtype antigens. Antibodies to A) subtype C CN54rgp140, B) subtype B gp160 and C) CRF01_AE gp120 were determined at three time points; four weeks after three HIV-DNA and two HIV-MVA vaccinations, and four weeks after the first and second CN54rgp140/GLA-AF immunization. Black dots show vaccinees who received 7 vaccinations, while colored dots show unprimed vaccinees who received two CN54rgp140/GLA-AF immunizations only. The Mann-Whitney test was used for statistical comparisons.

Fig 2 shows binding IgG antibody responses against subtype C, B and CRF01_AE Env measured four weeks after three HIV-DNA and two HIV-MVA vaccinations and four weeks after the first and second CN54rgp140/GLA-AF immunizations, respectively.

After three HIV-DNA and two HIV-MVA vaccinations, 34/35 (97%) vaccinees exhibited subtype C CN54rgp140 antibodies with a median titer of 900 (range 100–8100) in responders. All 35 (100%) vaccinees exhibited antibodies to subtype C CN54rgp140 after the first and second CN54rgp140/GLA-AF vaccination with median titers of 8100 (range 300–72900) and 24300 (range 2700 to > 72900), respectively. The magnitude of antibody responses to subtype C Env was significantly higher both after the first and second CN54rgp140/GLA-AF immunization compared to after the second HIV-MVA immunization, p<0.0001. After the first Env protein immunization, 2 of 5 unprimed vaccinees mounted an antibody response to subtype C CN54rgp140. The titers to subtype C Env in the two unprimed responding vaccinees were 100 and 8100. After the second Env protein boost 4/4 unprimed vaccinees exhibited anti-CN54rgp140 antibodies with a median titer of 5400 (range 2700–24300). The median titers in vaccinees who had received three HIV-DNA and two HIV-MVA immunizations followed by two CN54rgp140/GLA-AF immunizations were not significantly different from the median titer in those who were not primed before receipt of two CN54rgp140/GLA-AF immunizations (p = 0.077).

The absolute concentration of CN54rgp140-specific IgG was measured at three time points (S1 Fig). The absolute concentrations echoed what was seen when determining end point titers. The concentration of CN54rgp140 antibodies increased significantly from a median of 0.861 (range 0.197–4.449 μg/ml) after the HIV-DNA and HIV-MVA vaccinations to a median of 9.866 (range 0.134–129.6 μg/ml) after the first dose of CN54rgp140/GLA-AF (p<0.001) and then increased further to a median of 17.75 μg/ml (range 0.277 to 219.8 μg/ml) after the second Env protein immunization (p<0.001). The increase between the two boosts was statistically significant (p = 0.012).

After three HIV-DNA and two HIV-MVA vaccinations, 25/30 (87%) of evaluable vaccinees had detectable antibodies to subtype B gp160 with a median titer of 400 (range 100–1600) in responders. All 30 (100%) vaccinees exhibited antibodies to subtype B gp160 after the first CN54rgp140/GLA-AF vaccination with a median titer of 2700 (range 100–72900). After the second subtype C rgp140/ GLA-AF vaccination, 28/29 (97%) of vaccinees had subtype B gp160 antibodies with a median titer of 2700 (range 300–72900) (Fig 2). The magnitude of antibody responses to subtype B gp160 was significantly higher both after the first and second CN54rgp140/GLA-AF immunization than after the second HIV-MVA immunization, p<0.0001. A significant correlation was demonstrated between HIV-1 subtype C CN54rgp140 antibody titers and subtype B gp160 antibody titers after the first and the second CN54rgp140/GLA-AF vaccinations r = 0.7 (p<0.0001) and r = 0.59 (p = 0.0005), respectively (S2 Fig).

None of the five TaMoVac 01 placebo recipients had antibodies to subtype B gp160 before the CN54rgp140/GLA-AF vaccination. Two of the unprimed volunteers exhibited subtype B gp160-binding antibodies after the first and second CN54rgp140/GLA-AF vaccination.

After three HIV-DNA and two HIV-MVA vaccinations, 17/35 (49%) vaccinees exhibited subtype E gp120-specific antibodies which increased to 24/35 (69%) after the first CN54rgp140/GLA-AF immunization, p = 0.144. After the second CN54rgp140/GLA-AF immunization the proportion of responders, 28/35 (80%) was significantly higher as compared to that after the HIV-DNA and HIV-MVA immunization, p = 0.012.

The median titer of subtype E gp120-specific antibodies among the vacinees who had received three HIV-DNA and two HIV-MVA vaccinations was 900 (range 100–2700). The magnitude of subtype E gp120-specific antibodies was similar after the first and the second CN54rgp140/GLA-AF immunization with a median titer of 900 (range 100–8100) at both time points. One of the unprimed vaccinees exhibited subtype E gp120-specific antibodies with low titers (100) after the first and second CN54rgp140/GLA-AF immunization.

We did not see an influence on the binding antibody titers by time interval between the last HIV-MVA vaccination and the first CN54rgp140/GLA-AF boost, although the time interval varied considerably (31–70 weeks) between vaccinees (S3 Fig).

#### Functional antibody responses (Neutralizing Ab and ADCC)

Neutralization and ADCC-mediating antibody responses were assessed at two time points; after the second HIV-MVA/placebo and after the second CN54rgp140/GLA-AF immunization (Table 2). None of the 35 vaccinees who previously received HIV-DNA and HIV-MVA had positive responses in the pseudovirus/TZM-bl assay against SF 162 subtype B, GS015 subtype C or CM235 CRF01_AE. Using the IMC/PBMC assay, 7/35 (20%) vaccinees had neutralizing activity against GS015 subtypes C after the second HIV-MVA, while 11/35 (31%) had positive responses after the second CN54rgp140/GLA-AF immunization (p = 0.4125). Neutralizing antibody activity in the IMC/PBMC assay against CM235 CRF01_AE was seen in two of 35 vaccinees after the second HIV-MVA as well as after the second CN54rgp140/GLA-AF immunization. ADCC-mediating antibodies against CM235 CRF01_AE were demonstrated in 10 of 35 (29%) vaccinees four weeks after the second HIV-MVA immunization and in 9 of 35 (25%) vaccinees four weeks after the second CN54rgp140/GLA-AF immunization.

**Table 2:** Frequency of neutralization and ADCC antibody responses after the second HIV-MVA and after the second CN54rgp140/GLA-AF immunization.

Assay	IMC	Subtypes	Time point^a^ and number of positive/number tested (%)		
			After 2^nd^ MVA	After 2^nd^ CN54rgp140/GLA-AF	After 2 CN54rgp140/GLA-AF only
NAb, pseudovirus/ TZM-bl cells	SF 162	B	0/35(0%)	0/35(0%)	0/4
	GS015	C	0/35(0%)	0/35(0%)	0/4
	CM235	CRF01_AE	0/35(0%)	0/35(0%)	0/4
NAb, IMC/PBMC	SF 162	B	1/35 (3%)	0/35 (0%)	0/4
	GS015	C	7/35 (20%)	11/35(31%)	0/4
	CM235	CRF01_AE	2/35(6%)	2/35 (6%)	1/4
ADCC, IMC-LucR infected CEM.NKR_CCR5_	SF162	B	2/35 (6%)	1/35 (3%)	0/4
	1086	C	0/35 (0%)	1/35 (3%)	0/4
	CM 235	CRF01_AE	10/35 (29%)	9/35 (28%)	0/4

IMC: Infectious Molecular Clone; NAb: neutralizing antibody

^a^ four weeks after each of the indicated time points

**Fig 3:**
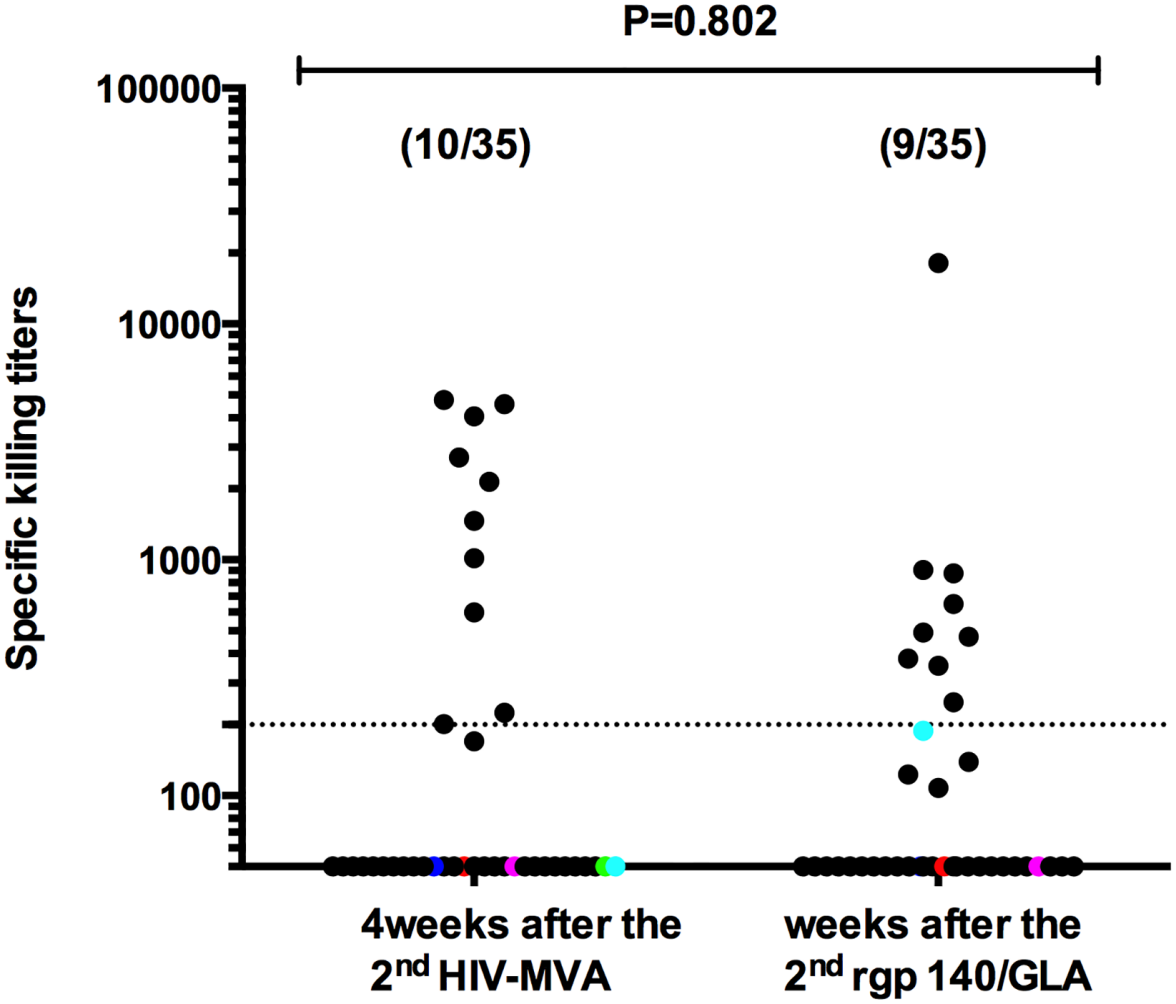
ADCC-mediating responses to CM235 CRF01_AE infected IMC. ADCC mediating antibodies four weeks after receipt of three HIV-DNA and two HIV-MVA vaccinations and four weeks after two CN54rgp140/GLA-AF immunizations are shown. Black dots show vaccinees who received 7 vaccinations, while colored dots show unprimed vaccinees who received two CN54rgp140/GLA-AF immunizations only. The Mann-Whitney test was used for statistical comparison.

The magnitude of the ADCC-mediating antibody response against CM235 CRF01_AE did not differ significantly between the two time points (Fig 3). The median titer four weeks after the second HIV-MVA was 1801 (range 201–4772) and four weeks after the second CN54rgp140/GLA-AF median titer was 491 (range 249–18140) p = 0.802. Only one of 35 vaccinees developed ADCC-mediating antibody to HIV 1086.c subtype C IMC after the second CN54rgp140/GLA-AF vaccination.

One of four volunteers who was unprimed prior to the two subtype C CN54rgp140/GLA-AF immunizations had neutralizing activity against CM235 CRF01_AE after the second CN54rgp140/GLA-AF immunization in the IMC/PBMC assay, yet none developed ADCC-mediating antibody responses after two CN54rgp140/GLA-AF immunizations.

#### IFN-γ ELISpot responses

**Fig 4:**
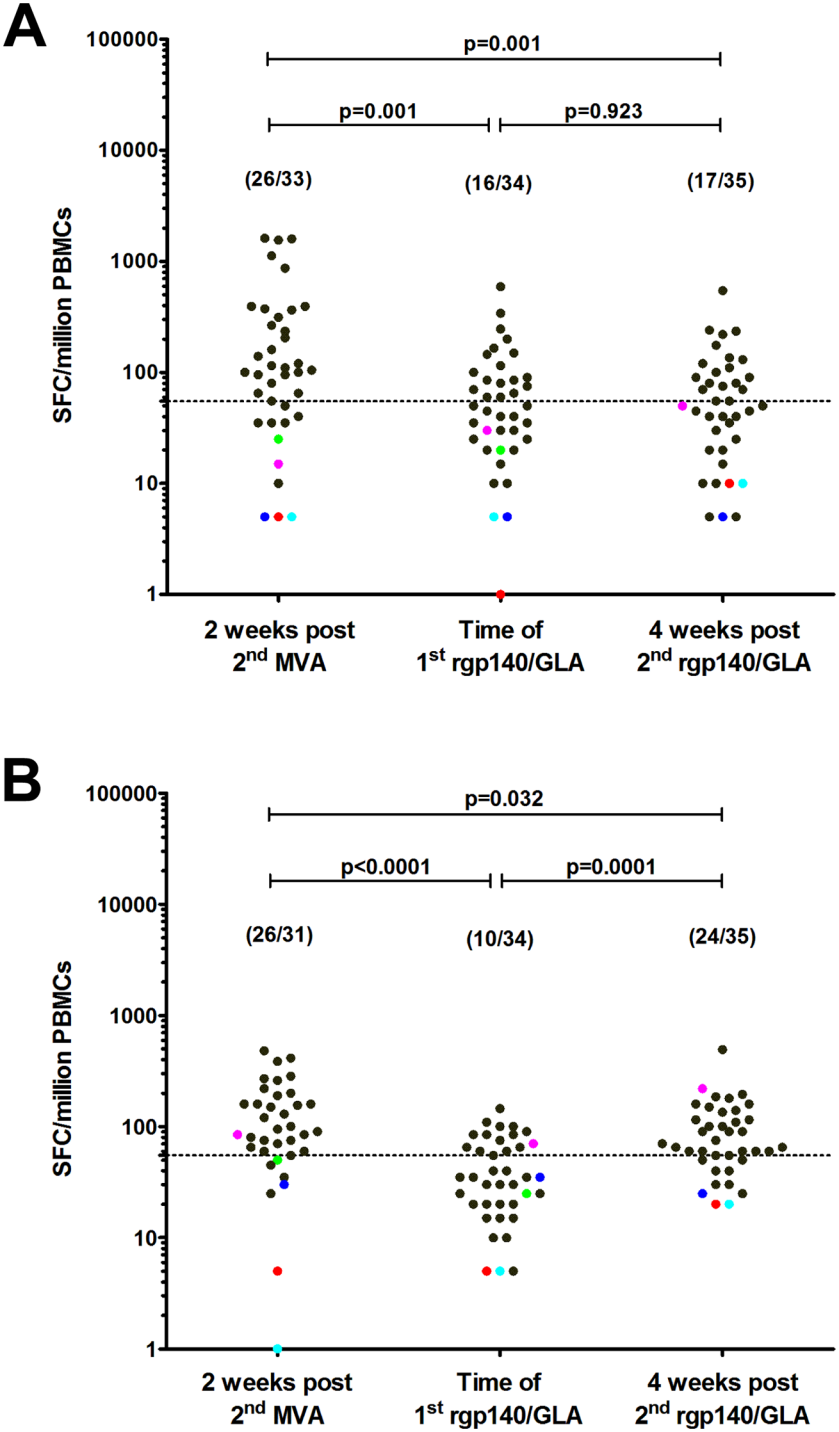
Magnitude of the IFN-γ ELISpot responses. IFN-γ ELISpot to (A) Gag and (B) the Env peptide pool after the second HIV-MVA, at the time of first CN54rgp140/GLA-AF and four weeks after the second CN54rgp140/GLA-AF immunizations. ELISpot responses were considered positive if the number of spot-forming cells (SFC) was > 55 spots/million peripheral blood mononuclear cells (PBMCs) and four times the background value. Black dots show vaccinees who received 7 vaccinations and colored dots show volunteers who received two CN54rgp140/GLA-AF immunizations only. The number of IFN-γ ELISpot responders per number of tested volunteers are given in parentheses. The Mann-Whitney test was used to compare the magnitude at different time points.

Fig 4 shows the IFN-γ ELISpot responses. The IFN-γ ELISpot responses were assessed in 35 volunteers who had received all seven vaccinations and in volunteers who received placebo and two CN54rgp140/GLA-AF vaccinations. Two weeks after the second HIV-MVA, 26/33 (79%) of the evaluable vaccinees had IFN-γ ELISpot responses to Gag-CMDR and 26/31 (84%) to Env-CMDR. At the time of the first CN54rgp140/GLA-AF immunization, 16/34 (47%) vaccinees still had IFN-γ ELISpot to Gag-CMDR and 10/34 (29%) to Env CMDR. Four weeks after the second CN54rgp140/GLA-AF immunization, the IFN-γ ELISpot response rate to Gag-CMDR was 17/35 (49%), similar to what was seen at the time of the first CN54rgp140 immunization. In contrast, the response rate to Env had increased significantly to 24/35 (69%), p = 0.0017, relative to the response seen at the time of the first CN54rgp140/GLA-AF immunization but was similar to the level seen after the second HIV-MVA vaccination (Fig 4). None of the vaccinees responded to the Pol-CMDR peptide pool at any of the three time points. Two weeks after the second HIV-MVA vaccination, the median response to Gag-CMDR was 182 SFC/million PBMCs (range 65–1610) and to Env-CMDR 140 SFC/million PBMCs (range 60–480). There was no significant difference between the magnitude of responses to Gag-CMDR among the responders at the time of the first CN54rgp140/GLA-AF, median 95 SFC/ million PBMCs (range 60–590) and four weeks after the second CN54rgp140/GLA-AF immunization, median 110 SFC/million PBMCs (range 70–545), p = 0.87. Interestingly, the magnitude of responses to Env-CMDR was significantly higher four weeks after the second CN54rgp140/GLA-AF than at the time of the first CN54rgp140/GLA-AF immunization, median 100 SFC/million PBMCs (range 60–495) versus 85 (range 60–145) p<0.0001.

One of the four (25%) unprimed vaccinees had a response to Env-CMDR at the time of (70 SFC/million PBMCs) and after the second CN54rgp140/GLA-AF immunization (220 SFC/million PBMCs. We did not see an influence on the magnitude of IFN-γ ELISpot responses by time interval between the last HIV-MVA vaccination and the first CN54rgp140/GLA boost, although the time interval varied considerably (31–70 weeks) between vaccinees (S3 Fig).

#### Lympoproliferative responses

**Fig 5:**
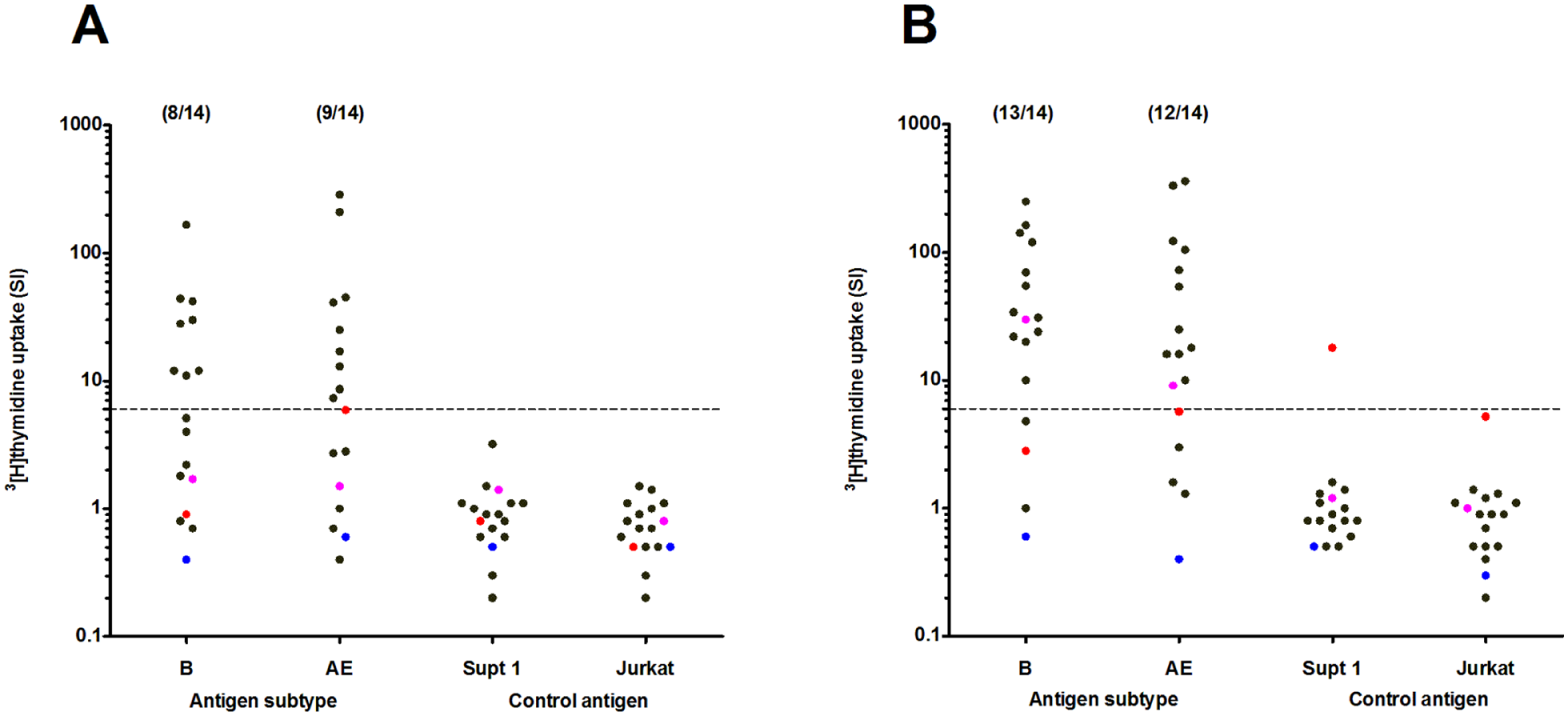
Magnitude of the lymphoproliferative responses. Responses at A) the time of the first CN54rgp140/GLA-AF immunization and B) four weeks after the second CN54rgp140/GLA-AF immunization to AT-2 treated subtype B MN and CRF01_AE antigen and their respective control antigen (Supt1 and Jurkat) are shown. Black dots show vaccinees who received 7 vaccinations and colored dots show volunteers who received two CN54rgp140/GLA-AF immunizations only. The number of vaccinees with a positive lymphoproliferative response per number of tested volunteers are given in parentheses. The Mann-Whitney test was used for statistical comparisons.

Lymphoproliferative responses are shown in Fig 5. Proliferative responses to AT-2 treated HIV MN subtype B and HIV CM235 CRF01_AE antigen were measured in 19 vaccinees from the MUHAS trial site, three of whom were placebo recipients before the two CN54rgp140/GLA-AF immunizations. The frequency of LPA response to subtype B MN at the time of the first CN54rgp140/GLA-AF immunization among the evaluable vaccinees who had received three HIV-DNA and two HIV-MVA immunizations was 8/14 (57%) which increased to 12/14 (86%) two weeks after the second CN54rgp140/GLA-AF, p = 0.442. The frequency of responses to CRF01_AE CM235 was 9/14(64%) at the time of first CN54rgp140/GLA-AF immunization which increased to 11/14 (79%) two weeks after the second CN54rgp140/GLA-AF, p = 0. 677. The magnitude of the LPA response among the responders increased significantly against subtype B MN from the time of the first CN54rgp140/GLA-AF immunization, median SI 29 (range 11–166) to after the second rgp140/GLA-AF immunization, median SI 34 (range 10–250) p = 0.041. None of the three unprimed vaccinees had LPA responses at the time of the first CN54rgp140/GLA-AF immunization, but after receipt of two CN54rgp140/GLA-AF immunizations one vaccinee developed LPA responses to subtype B MN and CRF01_AE CM235.

## Discussion

To our knowledge, this is the first clinical study of an HIV vaccine to combine DNA, MVA and adjuvanted protein. We were able to conduct the study as an amendment to a phase IIa trial in which healthy Tanzanian volunteers received three immunizations ID with HIV-DNA/placebo followed by two immunizations with HIV-MVA/placebo IM [16]. Forty volunteers were recruited to receive two additional immunizations with subtype C CN54rgp140 envelope protein adjuvanted with GLA-AF 31–70 weeks after the last HIV-MVA/placebo and 39 received both immunizations. The adjuvanted Env protein was well tolerated, except in one patient with pre-existing hypoglycaemia who experienced a clinically asymptomatic, transient aggravation of severe hypoglycaemia. Severe hypoglycaemia related to the CN54rgp140 has not been reported in the previous phase I trials using this protein [17, 18], but has been speculated to be associated with some vaccines, especially in metabolically vulnerable individuals [28,29]. Thus vaccine-induced hypoglycaemia warrants further study in future trials.

In the current study both humoral and cellular immune responses were boosted by CN54rgp140/GLA-AF immunizations. Systemic binding antibodies to CN54rgp140 increased significantly after each vaccination and reached titers of a median 23400. In the RV144 trial, in which the AIDSVax gp120 Env protein in alum was given twice together with ALVAC after “priming” twice with ALVAC alone, the estimated geometric mean antibody titer to gp120 subtype B MN was 31207 and to gp120 subtype E A244 was 14558 [7]. In the present study, binding antibody responses to subtype B gp160 and subtype E gp120 Env proteins were also significantly boosted by the CN54rgp140/GLA-AF protein but only in response to the first and not the second immunization. The effect of the adjuvanted gp140 Env protein on functional antibody responses was, however, disappointing with no detectable impact on IMC/PBMC neutralization or ADCC responses. The DNA and MVA envelope inserts did not match the CN54rgp140 although one DNA envelope plasmid was also subtype C. Optimal prime for CN54rgp140 boost may be a sequence matched envelope insert for DNA and/or MVA. This combination is not available in cGMP vaccine for human use. It is worth noting that the frequency of ADCC-mediating antibody responses in the present TaMoVac 01 trial was lower than reported in our previous phase I HIVIS03 trial where Tanzanian vaccinees received three HIV-DNA immunizations either 1 mg ID or 3.8 mg IM followed by two HIV-MVA vaccinations IM [13,15].

The RV144 trial reported modest efficacy. Similar to what is described here, binding antibodies to Env were seen in 98% of individuals [7]. Extensive post-hoc analyses of the RV144 trial generated two correlates; antibodies to Env V1/V2 scaffolded protein correlated with reduced infection risk, whereas the levels of monomeric IgA Env-specific binding antibodies correlated with increased risk. In the presence of low levels of Env-specific IgA, ADCC-activity was inversely correlated with risk of infection, suggesting that IgA might interfere with protective antibody function, presumed to be mediated via ADCC [8]. The same Env vaccine (gp120 AIDSVAX B/E) and adjuvant (alum) were used in the VAX003 trial, which reported no efficacy [3]. The authors suggested that the seven repeated relatively high doses of 600 μg of gp120 given in VAX003 in comparison to the four doses given in the RV144 trial may have had some bearing on the different balance in the distribution of antibody isotypes seen, which is assumed to be relevant to protection [30]. There has been much speculation around whether the co-administration of AIDSVAX B/E and ALVAC canarypox vaccine contributed to the functional antibody responses and this remains a possibility. In the recently completed TaMoVac II phase IIa trial, 198 volunteers in Tanzania and Mozambique have been randomized to receive three ID HIV-DNA immunizations given with or without electroporation followed by two HIV-MVA immunizations given with or without CN54rgp140 protein boosts in a factorial design.

In the present study there were increases both in the frequency and magnitude of Env-specific IFN-γ ELISpot responses and T-cell proliferative responses to AT-2 treated subtype B MN and CRF01_AE CM235 antigen after the CN54rgp140/ GLA-AF boosts. We have previously shown the HIV-DNA and HIV-MVA vaccines to be particularly potent T-cell and B-cell immunogens resulting in strong balanced Gag and Env-specific T-cell responses [13,14].

This study has some limitations. We did not measure antibody responses by isotype, nor antibody responses to scaffolded Env V1/V2 protein, and so opportunities for direct comparison with findings in the RV144 trial are limited. The titer of binding Ab which we observed was in the same range as that reported in the RV144 trial. The antibody avidity was not assessed after recombinant protein boost in this study due to limitations in funding but we know from our previous study that the DNA/MVA regimen could increase the avidity of induced antibodies [14].

There was considerable variation in the time between the last HIV-MVA and the first CN54rgp140 boost (8–17 months) but this did not appear to affect immune response results. After two CN54gp140/GLA-AF immunizations, we found a trend towards higher median anti-CN54 rgp140 subtype C titers in vaccinees previously immunized with three HIV-DNA and two HIV-MVA vaccinations (n = 35) compared to the four unprimed vaccinees who had received placebo five times before the CN54rgp140/GLA-AF immunizations (p = 0.07). The findings are inconclusive due to the low number of unprimed vaccinees. The recruitment into this amendment of the protocol was on a first come first serve basis and the number of volunteers having received placebo vaccinations instead of HIV-DNA and HIV-MVA was low. Therefore, only five placebo recipients were recruited and received CN54rgp140/GLA-AF immunizations.

In conclusion, we report significantly enhanced and broad binding antibody responses, and boosted Env-specific cell-mediated immune responses after two immunizations with a subtype C rgp140 protein adjuvanted in GLA-AF in individuals previously immunized with HIV-DNA and HIV-MVA, but no boosting of functional antibody responses.

## Supporting Information

S1 CONSORT Checklist (PDF)

S1 Fig The absolute concentration of CN54rgp140-specific IgG measured at three time points. (TIF)

S2 Fig A positive correlation between HIV-1 subtype C CN54 anti-gp140 and HIV-1 subtype B IIIB anti-gp160 titers. The figure shows findings at A) four weeks after receipt of three HIV-DNA and two HIV-MVA vaccinations, B) four weeks after the first CN54rgp140/GLA-AF boost and C) four weeks after the second CN54rgp140/GLA-AF boost. Thirty data points are included at each timepoint. Due to overlap all are not shown. The volunteers that were placebo recipients before the rgp140 boosting and five volunteers with invalid anti-gp160 results were excluded. Correlation was determined using Spearman rank correlation method.
(TIF)

S3 Fig Antibody binding responses and IFN-γ ELISpot responses by time interval between the second HIV-MVA vaccination and the first CN54rgp140/GLA-AF immunization. The figure shows the subtype B gp160 ELISA titers at the time of CN54rgp140/GLA-AF immunization(A), subtype B gp160 ELISA titers 2 weeks after the CN54rgp140/GLA-AF immunization (B), subtype C gp140 ELISA titers at the time of CN54rgp140/GLA-AF immunization(C), subtype C gp140 ELISA titers 2 weeks after the CN54rgp140/GLA-AF immunization (D), Gag-specific IFN-γ ELISpot responses at the time of CN54rgp140/GLA-AF immunization (E), Gag-specific IFN-γ ELISpot responses 2 weeks after the CN54rgp140/GLA-AF immunization (F), Env-specific responses at the time of CN54rgp140/GLA-AF immunization (G) and Env-specific IFN-γ ELISpot responses 2 weeks after the CN54rgp140/GLA-AF immunization (H).
(TIF)

S1 Protocol (DOC)

S1 Table HIV-1 antigens represented in prime-boost vaccines. (DOCX)

S2 Table Baseline characteristics of study participants by previous vaccine groups in TaMoVac 01. (DOCX)

S3 Table Numbers and percentages of clinical adverse events, solicited local and systemic reaction, and maximum new or worsened laboratory adverse events after immunization experienced per participants. (DOCX)
