# Supplementary material for: Boosting with Subtype C CN54rgp140 Protein Adjuvanted with Glucopyranosyl Lipid Adjuvant after Priming with HIV-DNA and HIV-MVA Is Safe and Enhances Immune Responses: A Phase I Trial
Source: PLoS One. 2016 May 18;11(5):e0155702. doi: 10.1371/journal.pone.0155702 (PMC4871571; doi:10.1371/journal.pone.0155702)
Supplement: S1 CONSORT Checklist — (PDF) [file pone.0155702.s001.pdf]

CONSORT 2010 checklist of information to include when reporting a randomised trial*

Section/Topic	Item No	Checklist item	Reported on page No
**Title and abstract**			
	1a	Identification as a randomised trial in the title	Not applicable
	1b	Structured summary of trial design, methods, results, and conclusions (for specific guidance see CONSORT for abstracts)	Structured abstract on page 3,4
**Introduction**			
Background and objectives	2a	Scientific background and explanation of rationale	Pages 5,6
	2b	Specific objectives or hypotheses	Page 6,7
**Methods**			
Trial design	3a	Description of trial design (such as parallel, factorial) including allocation ratio	Page 7,8 Table 1
	3b	Important changes to methods after trial commencement (such as eligibility criteria), with reasons	Not applicable
Participants	4a	Eligibility criteria for participants	Page 7,8
	4b	Settings and locations where the data were collected	Page 7,8
Interventions	5	The interventions for each group with sufficient details to allow replication, including how and when they were actually administered	Pages 7, 8,9 and table 1
Outcomes	6a	Completely defined pre-specified primary and secondary outcome measures, including how and when they were assessed	Page 15
	6b	Any changes to trial outcomes after the trial commenced, with reasons	Not applicable
Sample size	7a	How sample size was determined	Not applicable
	7b	When applicable, explanation of any interim analyses and stopping guidelines	Not applicable
Randomisation:			
Sequence generation	8a	Method used to generate the random allocation sequence	Not applicable
	8b	Type of randomisation; details of any restriction (such as blocking and block size)	Not applicable
Allocation concealment mechanism	9	Mechanism used to implement the random allocation sequence (such as sequentially numbered containers), describing any steps taken to conceal the sequence until interventions were assigned	Not applicable

Implementation	10	Who generated the random allocation sequence, who enrolled participants, and who assigned participants to interventions	Not applicable
Blinding	11a	If done, who was blinded after assignment to interventions (for example, participants, care providers, those assessing outcomes) and how	Not applicable
	11b	If relevant, description of the similarity of interventions	Not applicable
Statistical methods	12a	Statistical methods used to compare groups for primary and secondary outcomes	Not applicable
	12b	Methods for additional analyses, such as subgroup analyses and adjusted analyses	Not applicable
**Results**			
Participant flow (a diagram is strongly recommended)	13a	For each group, the numbers of participants who were randomly assigned, received intended treatment, and were analysed for the primary outcome	Figure 1
	13b	For each group, losses and exclusions after randomisation, together with reasons	Figure 1
Recruitment	14a	Dates defining the periods of recruitment and follow-up	Page 16 and 17
	14b	Why the trial ended or was stopped	Not applicable
Baseline data	15	A table showing baseline demographic and clinical characteristics for each group	S2 Table
Numbers analysed	16	For each group, number of participants (denominator) included in each analysis and whether the analysis was by original assigned groups	Page 16, 17, 18, 19, 20, 21,22,23,24, Table 2
Outcomes and estimation	17a	For each primary and secondary outcome, results for each group, and the estimated effect size and its precision (such as 95% confidence interval)	Page 17, 20,22,23
	17b	For binary outcomes, presentation of both absolute and relative effect sizes is recommended	Not applicable
Ancillary analyses	18	Results of any other analyses performed, including subgroup analyses and adjusted analyses, distinguishing pre-specified from exploratory	Not applicable
Harms	19	All important harms or unintended effects in each group (for specific guidance see CONSORT for harms)	Page 16<br>S3 Table
**Discussion**			
Limitations	20	Trial limitations, addressing sources of potential bias, imprecision, and, if relevant, multiplicity of analyses	Page 26
Generalisability	21	Generalisability (external validity, applicability) of the trial findings	Page 27
Interpretation	22	Interpretation consistent with results, balancing benefits and harms, and considering other relevant evidence	Not applicable
**Other information**			
Registration	23	Registration number and name of trial registry	WHO

			International Clinical Trial Registry PACTR20100 50002122368
Protocol	24	Where the full trial protocol can be accessed, if available	Attached supporting documents S1 Protocol
Funding	25	Sources of funding and other support (such as supply of drugs), role of	Attached separately in the online submission on sub section Funding statement

*We strongly recommend reading this statement in conjunction with the CONSORT 2010 Explanation and Elaboration for important clarifications on all the items. If relevant, we also recommend reading CONSORT extensions for cluster randomised trials, non-inferiority and equivalence trials, non-pharmacological treatments, herbal interventions, and pragmatic trials. Additional extensions are forthcoming: for those and for up to date references relevant to this checklist, see www.consort-statement.org.
