# Supplementary figures and images for: Boosting with Subtype C CN54rgp140 Protein Adjuvanted with Glucopyranosyl Lipid Adjuvant after Priming with HIV-DNA and HIV-MVA Is Safe and Enhances Immune Responses: A Phase I Trial
Source: PLoS One. 2016 May 18;11(5):e0155702. doi: 10.1371/journal.pone.0155702 (PMC4871571; doi:10.1371/journal.pone.0155702)

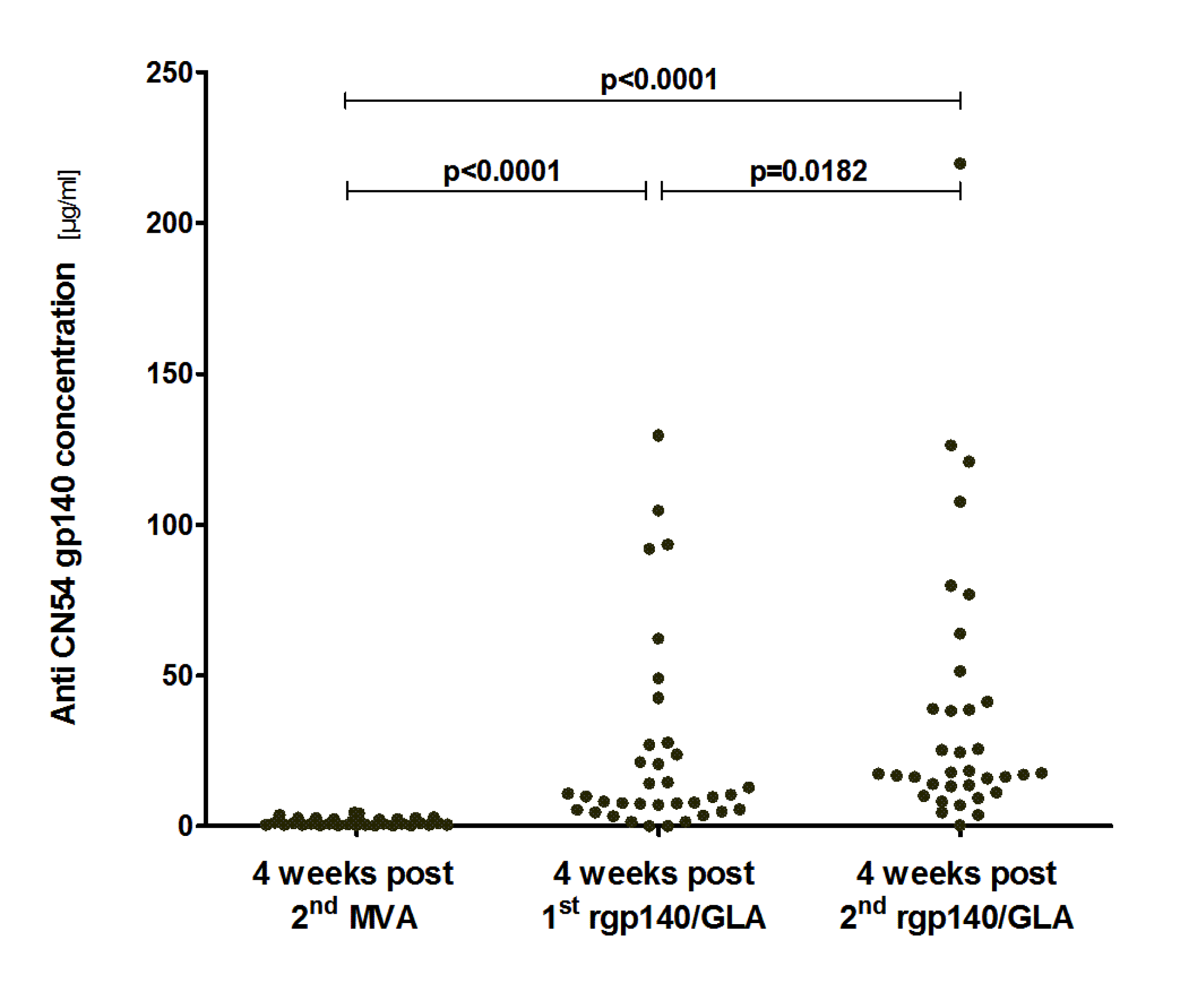

Supplement: S1 Fig — (TIF) [file pone.0155702.s002.tif]

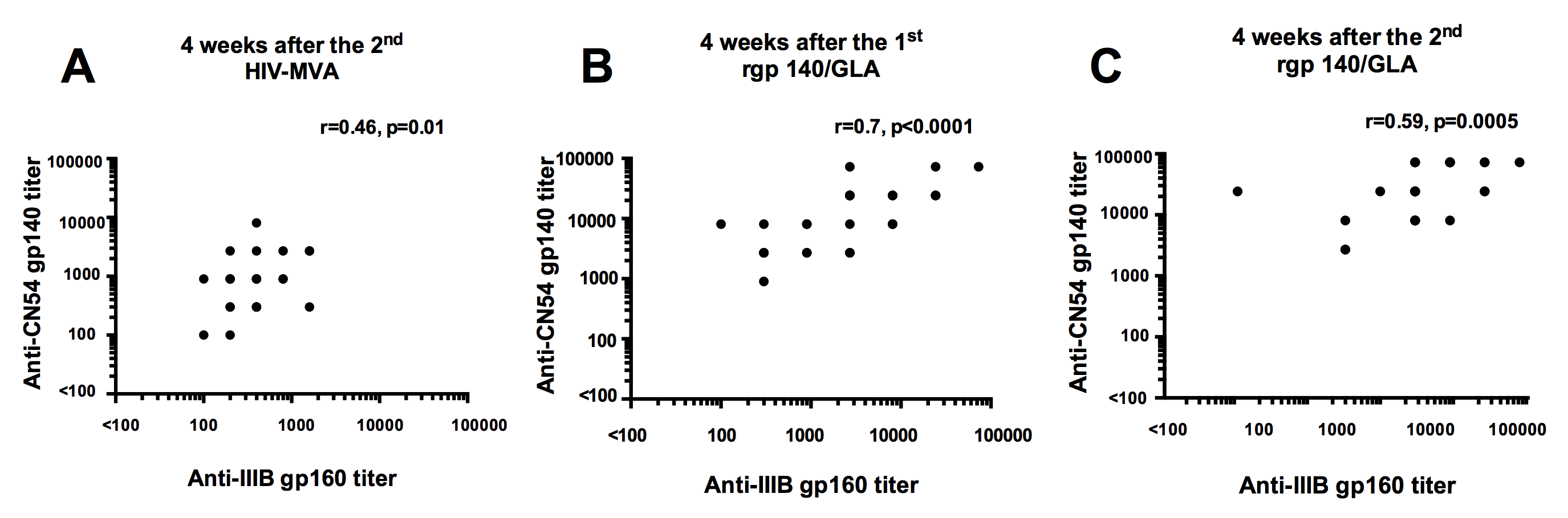

Supplement: S2 Fig — The figure shows findings at A) four weeks after receipt of three HIV-DNA and two HIV-MVA vaccinations, B) four weeks after the first CN54rgp140/GLA-AF boost and C) four weeks after the second CN54rgp140/GLA-AF boost. Thirty data points are included at each timepoint. Due to overlap all are not shown. The volunteers that were placebo recipients before the rgp140 boosting and five volunteers with invalid anti-gp160 results were excluded. Correlation was determined using Spearman rank correlation method. (TIF) [file pone.0155702.s003.tif]

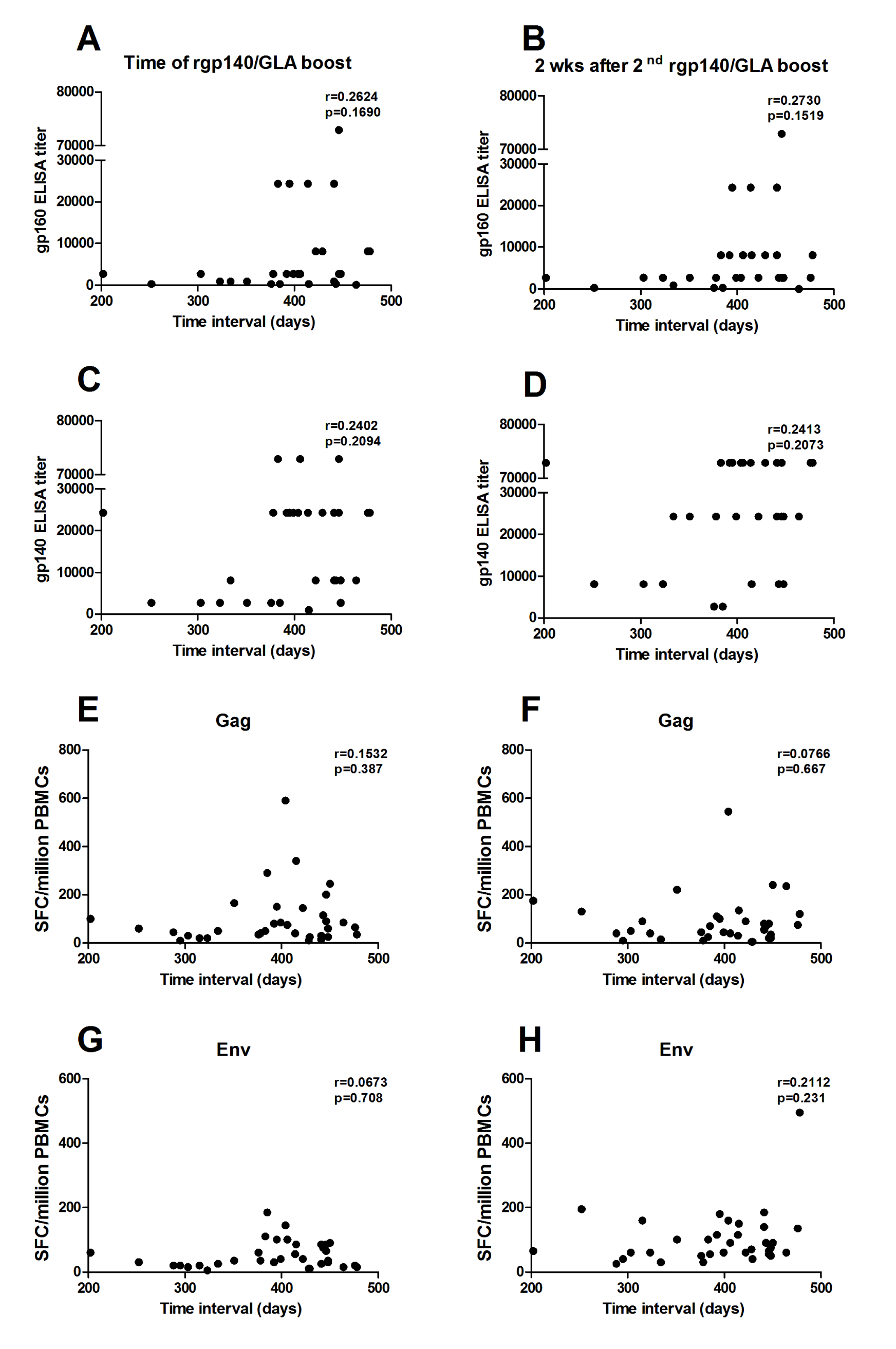

Supplement: S3 Fig — The figure shows the subtype B gp160 ELISA titers at the time of CN54rgp140/GLA-AF immunization(A), subtype B gp160 ELISA titers 2 weeks after the CN54rgp140/GLA-AF immunization (B), subtype C gp140 ELISA titers at the time of CN54rgp140/GLA-AF immunization(C), subtype C gp140 ELISA titers 2 weeks after the CN54rgp140/GLA-AF immunization (D), Gag-specific IFN-γ ELISpot responses at the time of CN54rgp140/GLA-AF immunization (E), Gag-specific IFN-γ ELISpot responses 2 weeks after the CN54rgp140/GLA-AF immunization (F), Env-specific responses at the time of CN54rgp140/GLA-AF immunization (G) and Env-specific IFN-γ ELISpot responses 2 weeks after the CN54rgp140/GLA-AF immunization (H). (TIF) [file pone.0155702.s004.tif]
