# Supplementary material for: Boosting with Subtype C CN54rgp140 Protein Adjuvanted with Glucopyranosyl Lipid Adjuvant after Priming with HIV-DNA and HIV-MVA Is Safe and Enhances Immune Responses: A Phase I Trial
Source: PLoS One. 2016 May 18;11(5):e0155702. doi: 10.1371/journal.pone.0155702 (PMC4871571; doi:10.1371/journal.pone.0155702)
Supplement: S1 Table — (DOCX) [file pone.0155702.s006.docx]

**S1 Table. HIV-1 antigens represented in prime-boost schedule.**

| **Vaccine component** | **HIV-1 protein expressed** | **HIV-1**  **subtype** | **Name, accession no.*** | **Vector** | **Ref.** |
| --- | --- | --- | --- | --- | --- |
| HIV-DNA  prime | Env gp160 | A1 | A1.UG92031, AY187680 | pkCMV | Ljungberg et al 2002 |
|  | Env gp160 | B | B.FR.HXB2, KO3455 | pkCMV | Ljungberg et al 2002 |
|  | Env gp160 | C | C.BR.92BR025, U52953 | pkCMV | Ljungberg et al 2002 |
|  | Gag p37 | A1/B | A1.UG92031  B.FR.HXB2 | pkCMV | Bråve et al 2005 |
|  | Gag p37 | B | B.FR.HXB2  KO3455 | pkCMV | Bråve et al 2005 |
|  | Rev | B | B.FR.HXB2  KO3455 | pkCMV | Kjerrström et al 2001 |
|  | RTmut | B | B.FR.HXB2  KO3455 | pkCMV | Isaguliants et al 2000 |
| HIV-MVA  boost | Env gp150 | E | CRF01_AE  CM235  AY736837 | MVA** | Earl et al 2009 |
|  | Gag p55 | A | CRF01_AE  CM240  US4771 | MVA | Earl et al 2009 |
|  | Pol (RTmut, PRmut) | A | CRF01_AE  CM240  US4771 | MVA | Earl et al 2009 |
| Gp140  boost | Env gp140*** | C | CN54gp140  97CN001 | None | Su et al 2000  Rodenburg et al 2001 |

*HIV database: <http://www.hiv.lanl.gov> **Modified Vaccinia Ankara MVAp579 ***Adjuvanted with Glucopyranosyl Lipid Adjuvant (GLA)

**References for S1 Table.**

Ljungberg K, Rollman E, Eriksson L, Hinkula J, Wahren B. [Enhanced immune responses after DNA vaccination with combined envelope genes from different HIV-1 subtypes.](http://www.ncbi.nlm.nih.gov/pubmed/12429515) Virology. 2002 Oct 10; 302(1):44-57.

Kjerrström A, Hinkula J, Engström G, Ovod V, Krohn K, Benthin R, et al. [Interactions of single and combined human immunodeficiency virus type 1 (HIV-1) DNA vaccines.](http://www.ncbi.nlm.nih.gov/pubmed/11352667) Virology. 2001 May 25; 284 (1): 46-61.

Isaguliants MG, Petrakova NN, Zuber B, Pokrovskaya K, Gizatullin R, Kostyuk DA, et al. [DNA-encoding enzymatically active HIV-1 reverse transcriptase, but not the inactive mutant, confers resistance to experimental HIV-1 challenge.](http://www.ncbi.nlm.nih.gov/pubmed/11251384) Intervirology. 2000; 43(4-6): 288-93.

# [Bråve A](http://www.ncbi.nlm.nih.gov/pubmed/?term=Br%C3%A5ve%20A%5BAuthor%5D&cauthor=true&cauthor_uid=16112909), [Ljungberg K](http://www.ncbi.nlm.nih.gov/pubmed/?term=Ljungberg%20K%5BAuthor%5D&cauthor=true&cauthor_uid=16112909), [Boberg A](http://www.ncbi.nlm.nih.gov/pubmed/?term=Boberg%20A%5BAuthor%5D&cauthor=true&cauthor_uid=16112909), [Rollman E](http://www.ncbi.nlm.nih.gov/pubmed/?term=Rollman%20E%5BAuthor%5D&cauthor=true&cauthor_uid=16112909), [Isaguliants M](http://www.ncbi.nlm.nih.gov/pubmed/?term=Isaguliants%20M%5BAuthor%5D&cauthor=true&cauthor_uid=16112909), [Lundgren B](http://www.ncbi.nlm.nih.gov/pubmed/?term=Lundgren%20B%5BAuthor%5D&cauthor=true&cauthor_uid=16112909), et al. Multigene/multisubtype HIV-1 vaccine induces potent cellular and humoral immune responses by needle-free intradermal delivery. [Mol Ther.](http://www.ncbi.nlm.nih.gov/pubmed/16112909) 2005 Dec;12(6):1197-205.

Earl PL, Cotter C, Moss B, VanCott T, Currier J, Eller LA, et al. D[esign and evaluation of multi-gene, multi-clade HIV-1 MVA vaccines.](http://www.ncbi.nlm.nih.gov/pubmed/19654066) Vaccine. 2009 Sep 25;27(42):5885-95.

Su L, Graf M, Zhang Y, von Briesen H, Xing H, Kostler J, et al. Characterization of a virtually full-length human immunodeficiency virus type 1 genome of a prevalent intersubtype (C/B') recombinant strain in China. J Virol. 2000;74(23):11367-76.

Rodenburg CM, Li Y, Trask SA, Chen Y, Decker J, Robertson DL, et al. Near full-length clones and reference sequences for subtype C isolates of HIV type 1 from three different continents. AIDS Res Hum Retroviruses. 2001;17(2):161-8.
