# Supplementary material for: Boosting with Subtype C CN54rgp140 Protein Adjuvanted with Glucopyranosyl Lipid Adjuvant after Priming with HIV-DNA and HIV-MVA Is Safe and Enhances Immune Responses: A Phase I Trial
Source: PLoS One. 2016 May 18;11(5):e0155702. doi: 10.1371/journal.pone.0155702 (PMC4871571; doi:10.1371/journal.pone.0155702)
Supplement: S2 Table — (DOCX) [file pone.0155702.s007.docx]

S2 Table. Baseline characteristics of study participants by previous vaccine groups in TaMoVac 01

| **Variable** | **Total** | **Group I** | **Group II** | **Group III** | **Placebo** |
| --- | --- | --- | --- | --- | --- |
|  | N=40 | N=10 | N=11 | N=14 | N=5 |
| Site |  |  |  |  |  |
| MMRC | 21 (52.5) | 5 (50.0) | 6 (54.6) | 8 (57.1) | 2 (40.0) |
| MUHAS | 19 (47.5) | 5 (50.0) | 5 (45.4) | 6 (42.9) | 3 (60.0) |
| Gender |  |  |  |  |  |
| female | 15 (37.5) | 4 (40.0) | 1 (9.1) | 8 (57.1) | 2 (40.0) |
| male | 25 (62.5) | 6 (60.0) | 10 (90.9) | 6 (42.9) | 3 (60.0) |
| Age | 25 (22-34) | 29 (23-35) | 26 (22-37) | 25 (21-34) | 22 (22-22) |
| Weeks between first DNA and first CN54gp140/GLA immunization | 103 (95-109) | 108 (103-112) | 101 (82-110) | 102 (90-106) | 107 (98-109) |
| Weeks between last MVA and first CN54gp140/GLA immunization | 60 (53-66) | 65 (61-66) | 58 (45-65) | 57 (52-62) | 64 (59-66) |
| **Laboratory** |  |  |  |  |  |
| Hemoglobin, (g/dL) | 14.9 (13.9-15.7) | 14.6 (13.9-15.6) | 15.1 (14.8-15.7) | 14.9 (13.0-16.0) | 14.6 (13.9-15.0) |
| White cell count, (10^9^ cells/L) | 4.7 (4.0-5.2) | 4.4 (4.0-5.0) | 4.4 (4.0-5.3) | 4.9 (4.6-5.2) | 4.2 (4.0-5.1) |
| Neutrophils, (10^9^ cells/L) | 2.1 (1.6-2.6) | 2.1 (1.5-2.4) | 2.0 (1.6-2.8) | 2.3 (1.8-2.8) | 1.7 (1.6-2.4) |
| Grade I | 1 (2.5) | 0 | 0 | 1 (7.1) | 0 |
| Grade IIII | 1 (2.5) | 0 | 0 | 1 (7.1) | 0 |
| Lymphocytes, (10^9^ cells/L) | 2.0 (1.7-2.3) | 1.9 (1.6-2.3) | 1.9 (1.6-2.3) | 2.2 (1.9-2.4) | 2.0 (1.9-2.1) |
| Platelets, (10^9^ cells/L) | 248 (212-287) | 232 (209-252) | 215 (188-262) | 280 (243-310) | 272 (230-284) |
| Grade I | 1 (2.5) | 0 | 1 (9.1) | 0 | 0 |
| ALT, (U/L) | 17 (14-26) | 17 (13-25) | 17 (15-27) | 21 (14-27) | 17 (11-18) |
| Grade I | 1 (2.5) | 0 | 0 | 1 (7.1) | 0 |
| Total Bilirubin, (µmol/l) | 7.9 (5.4-11.8) | 9.4 (7.5-11.7) | 7.7 (4.5-13.3) | 7.4 (6.3-11.9) | 5.4 (5.4-8.0) |
| Creatinine, (µmol/l) | 60 (53-74) | 67 (53-75) | 70 (58-75) | 57 (45-60) | 61 (53-75) |
| Glucose, (mmol/l) | 4.4 (4.0-4.9) | 4.5 (4.0-5.1) | 4.3 (3.9-4.6) | 4.6 (4.3-4.9) | 4.3 (3.8-4.5) |
| Grade I hypoglycaemia | 2 (5.0) | 1 (10.0) | 0 | 0 | 1 (20.0) |
| Grade II hypoglycaemia | 1 (2.5) | 0 | 1 (9.1) | 0 | 0 |
| Grade I hyperglycaemia | 1 (2.5) | 0 | 0 | 1 (7.1) | 0 |

Note: Values are numbers (%) or medians (interquartile ranges)
