# Supplementary material for: Boosting with Subtype C CN54rgp140 Protein Adjuvanted with Glucopyranosyl Lipid Adjuvant after Priming with HIV-DNA and HIV-MVA Is Safe and Enhances Immune Responses: A Phase I Trial
Source: PLoS One. 2016 May 18;11(5):e0155702. doi: 10.1371/journal.pone.0155702 (PMC4871571; doi:10.1371/journal.pone.0155702)
Supplement: S3 Table — (DOCX) [file pone.0155702.s008.docx]

**S3 Table. Numbers and percentages of clinical adverse events, solicited local and systemic reaction, and maximum new or worsened laboratory adverse events after immunization experienced per participants**

|  | **None** | **Mild** | **Moderate** | **Severe** |
| --- | --- | --- | --- | --- |
| **SOLICITED LOCAL REACTIONS** |  |  |  |  |
| Any local reaction, N (%) | 8 (20.0) | 29 (72.5) | 3 (7.5) | 0 |
| Pain, N (%) | 8 (20.0) | 29 (72.5) | 3 (7.5) | 0 |
| Swelling, N (%) | 25 (62.5) | 15 (37.5) | 0 | 0 |
| Warmth, N (%) | 33 (82.5) | 6 (15.0) | 1 (2.5) | 0 |
| Itching, N (%) | 34 (85.0) | 6 (15.0) | 0 | 0 |
| Papule, N (%) | 39 (97.5) | 1 (0.5) | 0 | 0 |
| Erythema/ Induration/ Blister, N (%) | 40 (100) | 0 | 0 | 0 |
| **SOLICITED SYSTEMIC REACTIONS** |  |  |  |  |
| Any systemic reaction, N (%) | 19 (47.5) | 19 (47.5) | 2 (5.0) | 0 |
| Headache, N (%) | 25 (62.5) | 14 (35.0) | 1 (2.5) | 0 |
| Malaise, N (%) | 28 (70.0) | 10 (25.0) | 2 (5.0) | 0 |
| Arthralgia, N (%) | 29 (72.5) | 10 (25.0) | 1 (2.5) | 0 |
| Myalgia, N (%) | 30 (75.0) | 9 (22.5) | 1 (2.5) | 0 |
| Nausea, N (%) | 37 (92.5) | 3 (7.5) | 0 | 0 |
| Vomiting, N (%) | 38 (95.0) | 2 (5.0) | 0 | 0 |
| Rigor, N (%) | 38 (95.0) | 2 (5.0) | 0 | 0 |
| **Clinical adverse Events** | 33 (82.5) | 5 (12.5) | 2 (5) |  |
| **LABORATORY EVENTS, HAEMATOLOGY** |  |  |  |  |
| Any haematology event, N (%) | 31 (77.5) | 6 (15.0) | 3 (7.5) |  |
| Anaemia, N (%) | 40 (100.0) | 0 | 0 | 0 |
| Leukopenia, N (%) | 38 (95.0) | 2 (5.0) | 0 | 0 |
| Neutropenia, N (%) | 33 (82.5) | 4 (10.0) | 3 (7.5) | 0 |
| Lymphopenia, N (%) | 40 (100.0) | 0 | 0 | 0 |
| Low platelets, N (%) | 38 (95.0) | 2 (5.0) | 0 | 0 |
| **LABORATORY EVENTS, BIOCHEMISTRY** |  |  |  |  |
| Any biochemistry event, N (%) | 27 (67.5) | 11 (27.5) | 1 (2.5) | 1 (2.5) |
| Creatinine elevation, N (%) | 40 (100.0) | 0 | 0 | 0 |
| ALT elevation, N (%) | 37 (92.5) | 3 (7.5) | 0 | 0 |
| Bilirubin elevation, N (%) | 40 (100.0) | 0 | 0 | 0 |
| Hypoglycaemia, N (%) | 31 (77.5) | 7 (17.5) | 1 (2.5) | 1 (2.5) |
| Hyperglycaemia, N (%) | 40 (100.0) | 0 | 0 | 0 |
